# Exploring the Role of Peritumoral Edema in Predicting Lung Cancer Subtypes Through T2-FLAIR Digital Subtraction Imaging of Metastatic Brain Tumors

**DOI:** 10.3390/diagnostics15101283

**Published:** 2025-05-20

**Authors:** Okan Dilek, Emin Demırel, Zeynel Abidin Tas, Emre Bılgın

**Affiliations:** 1 Department of Radiology, Adana City Training and Research Hospital, University of Health Sciences, 01370 Adana, Turkey; 2 Department of Radiology, Faculty of Medicine, Afyonkarahisar University of Health Sciences, 03030 Afyonkarahisar, Turkey; dremindemirel@gmail.com; 3 Department Pathology, Adana Teaching and Research Hospital, University of Health Sciences, 01230 Adana, Turkey; zeynelabidin46@gmail.com; 4 Department Neurosurgery, Adana Teaching and Research Hospital, University of Health Sciences, 01230 Adana, Turkey; dremreblgn@gmail.com

**Keywords:** digital subtraction imaging, small-cell lung cancer, non-small-cell lung cancer, radiomics, AI

## Abstract

**Background/Objectives**: This study aimed to investigate whether small-cell lung cancer (SCLC) and non-small-cell lung cancer (NSCLC) can be distinguished based on radiomics data derived from T2-FLAIR digital subtraction images of the peritumoral edema region in patients with brain metastases. **Methods:** A total of 136 patients who underwent surgery for brain tumors, including 100 patients in the Pretreat-Metstobrain-MASKS dataset and 36 patients from our institution, were included in our study. Radiomic features were extracted from digitally subtracted T2-FLAIR images in the peritumoral edema area. Patients were divided into NSCLC and SCLC groups. The maximum relevance–minimum redundancy (mRMR) method was then used for dimensionality reduction. The Naive Bayes algorithm was used for model development, and the interpretability of the model was explored using SHapley Additive exPlanations (SHAP). The performance metrics included the area under the curve (AUC), sensitivity (SENS), and specificity (SPEC). **Results:** The mean age of NSCLC patients was 64.6 ± 10.3 years, and that of SCLC patients was 63.4 ± 11.7 years. In the external validation cohort, the model achieved an AUC of 0.82 (0.68–0.97), a SENS of 0.87 (0.74–0.91), and a SPEC of 0.72 (0.72–0.89). In the train cohort, the model achieved an AUC of 1.000, a SENS of 1.000, and a SPEC of 1.000. The feature providing the best effect was wavelet-HHHglcmJointEnergy, with a SHAP value of approximately 2.5. **Conclusions:** An artificial intelligence model developed using radiomics data from T2-FLAIR digital subtraction images of the peritumoral edema area can identify the histologic type of lung cancer in patients with associated brain metastases.

## 1. Introduction

Lung cancer is a major global health problem, with an estimated 2 million new cases and more than 1.8 million deaths each year [1]. Furthermore, it is the cause of more than half of all solitary brain metastases, which lead to morbidity and mortality in lung cancer patients. Small-cell lung cancer (SCLC) and non-small-cell lung cancer (NSCLC) are two major types of lung cancer that exhibit significant differences in clinical features and treatment approaches. SCLC is a rapidly growing cancer that tends to metastasize early, typically spreading to the brain, liver, and bones [2]. Treatment for SCLC usually involves chemotherapy and radiotherapy, as the disease is often not amenable to surgical intervention and has a poor prognosis [3]. In contrast, NSCLC is a more heterogeneous cancer, divided into subtypes such as adenocarcinoma, squamous cell carcinoma, and large cell carcinoma [4]. The progression of NSCLC is generally slower, and better outcomes can be achieved with treatment methods such as surgical intervention, chemotherapy, and radiotherapy [5]. The prognosis of NSCLC is generally better than that of SCLC. SCLC has a higher incidence of brain metastasis compared to NSCLC [6,7]. The ability to distinguish between brain metastases of SCLC and NSCLC is crucial because SCLC tends to spread earlier in the course of the disease, has a more aggressive clinical course, and is typically managed with non-surgical treatments [8]. The biological and clinical differences between these two cancer types directly affect treatment approaches and patient prognoses.

While the diagnosis of lung cancer typically relies on the surgical resection of the primary lesion or metastases, it is not always possible to determine the histologic subtype from small biopsy or cytology samples obtained through diagnostic interventions. Therefore, differentiating the histologic subtype of lung cancer through imaging of brain metastases could be beneficial. However, among the current technical challenges are the difficulty in accurately determining histologic subtypes from limited biopsy samples and the difficulty in distinguishing the histologic subtypes of brain metastases. Nevertheless, it appears that radiomic data obtained from different magnetic resonance imaging (MRI) sequences hold potential in overcoming these challenges. Previous studies have examined the potential of radiomic data from various MRI sequences to distinguish histologic subtypes of lung cancer in brain metastases [9,10].

It has been shown that peritumoral edema can serve as an important marker of the response to radiotherapy and the prognosis of patients with lung cancer brain metastases [11,12]. The amount and content of peritumoral edema differ among brain metastases of various histologic subtypes of lung cancer. In the literature, different MR sequences have been used to attempt to differentiate histologic subtypes of brain metastases [13,14]. The artificial intelligence model developed using radiomic data obtained from T2-Fluid-Attenuated Inversion Recovery (FLAIR) digital subtraction images can differentiate between brain metastases and high-grade glial tumors [15]. The aim of this study is to investigate whether SCLC can be differentiated from NSCLC using radiomics data obtained from T2-FLAIR digital subtraction images of the peritumoral edema area in lung cancer patients with brain metastases.

## 2. Material and Methods

### 2.1. Patient Selection

This study utilized data from 100 lung cancer patients with brain metastases, sourced from the Pretreat-MetsToBrain-Masks dataset available in The Cancer Imaging Archive (TCIA) [16]. Image preprocessing was carried out as follows: For each patient, four standard conventional MRI sequences were co-registered with the SRl24 anatomical template. Subsequently, these sequences were resampled to achieve a uniform isotropic resolution of 1 mm (1 mm^3^) and were then skull stripped. The CaPTK program [17] was employed to automatically segment contrast-enhanced areas, necrosis, and peri-lesional edema areas for each patient. A neuroradiologist then meticulously reviewed and corrected the sequence and segmentation NIfTI files using the ITK-SNAP software (https://www.itksnap.org/). Patient characteristics, such as age, gender, pathological grade, and genomic profile, were retrieved from the TCIA. It is important to note that the TCIA dataset does not contain any personally identifiable information. Ethical approval and informed consent were obtained for the original study in which this open-source dataset was created. Images of patients from our institution were used in the validation cohort. In our institution, images were obtained on 1.5 T and 3 T Philips Ingenia (Philips Healthcare, Best, The Netherlands) devices. In our institution, axial non-contrast T1 and contrast-enhanced T1-weighted images, axial T2, and FLAIR images were obtained, similar to the acquisition protocols for the data obtained from the open-access brain MR protocol. We applied a similar preparation and segmentation process for the patients in our institution, and we used the CaPTK program for this purpose. The study was conducted in accordance with the Declaration of Helsinki, and the protocol was approved by the Ethics Committee of Adana City Training and Research Hospital Clinical Research (approval date: 11 September 2024; approval number: 165-2024). Based on the retrospective nature of this data analysis, detailed written informed consent of the patients and/or their chaperones for this study was waived.

The validation cohort was composed of images from patients within our institution. Our study received approval from the local ethics committee. We applied a similar preprocessing and segmentation procedure as that described above to our in-house patient data, utilizing the CaPTK program for this purpose as well. Inclusion criteria included a pathologically proven diagnosis of brain metastasis and availability of a pretreatment scan with standard MRI sequences (T1w, T1 post-gadolinium, T2w, and FLAIR). Exclusion criteria included lack of a pretreatment scan or one of the standard MR sequences, as well as significant motion artifacts in any of the standard sequences. The analysis focused on patients with primary lung cancer within the dataset. Three patients lacking a peritumoral edema area and five patients with poor image quality and image artifacts were excluded.

To ensure consistency with the TCIA data, similar parameters were also examined in patients obtained from our institution. Demographic data, including sex, age at diagnosis, and the presence of extranodal metastasis, were obtained from their electronic medical records (EMRs). Survival was calculated by subtracting the date of diagnosis from the date of death or from the date of the last EMR note for censored patients. Quantitative imaging features were extracted from the NifTI segmentation mask for each patient, including total enhancing tumor volume, total necrotic tumor volume, total peritumoral edema volume, ratio of necrotic to enhancing volume, ratio of peritumoral edema to enhancing volume, number of enhancing lesions, number of necrotic lesions, and number of lesions with peritumoral edema. Finally, the origin of metastasis was also obtained from the EMR according to previous oncological and/or pathological reports. Dates of birth, diagnosis, death, and last note were all anonymized, preserving the intervals between events.

Segmentation protocol: All segmentations were performed manually on DICOM images in a research PACS using a volumetric tool after transfer from the clinical PACS. All segmentations were approved by two radiologists.

**Figure 1:**
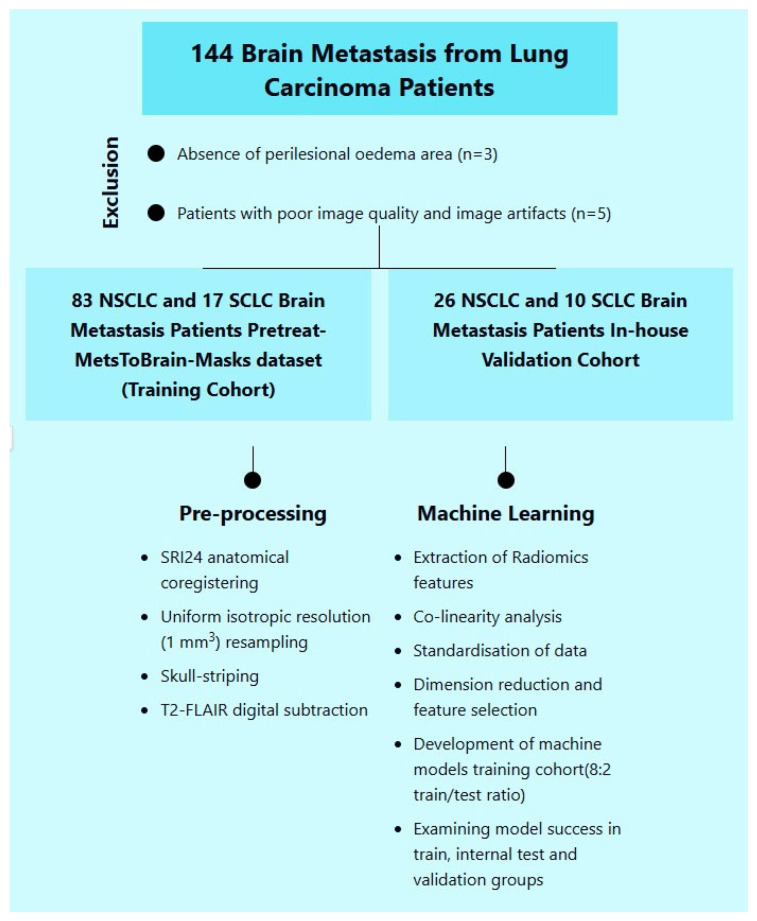
The numbers of included and excluded patients in the study.

Ultimately, a total of 136 patients were included in the study (Figure 1), comprising 27 patients with small-cell lung cancer (SCLC) and 109 with non-small-cell lung cancer (NSCLC). The study population was divided into training and validation cohorts. The training cohort consisted of 100 patients, including 17 with SCLC (17%) and 83 with NSCLC (83%). The validation cohort included 36 patients: 10 with SCLC (27.8%) and 26 with NSCLC (72.2%).

### 2.2. Generating T2-FLAIR Digital Subtraction Images

Initially, all patient and segmented images from the source dataset were co-registered and resampled to achieve isotropic resolution [18]. The datasets utilized in this study had undergone preprocessing steps, including signal normalization, resampling, co-registration, and skull stripping, for all four primary MRI sequences (T1, T1 + C, T2, and FLAIR). To perform digital subtraction of FLAIR images from the MR images, essential libraries such as nibabel and numpy were employed. The T2 and FLAIR images were converted into numpy arrays, and the FLAIR image was mathematically subtracted from the associated T2 image. The resulting difference image underwent normalization by scaling the image data between their minimum and maximum values. This normalized image was then saved in NIfTI format for subsequent analysis and visualization.

Three-dimensional (3D) rendering processes were implemented using the Python (https://www.python.org/) programming language and various open-source libraries. The SimpleITK library, which provides a comprehensive suite of medical image processing functions, was utilized for the manipulation of MR images and tumor masks. For conversion into 3D-rendered images, the Visualization Toolkit (VTK) and Matplotlib (https://matplotlib.org/stable/) libraries were employed, leveraging their capabilities for 3D data processing and visualization.

**Figure 2:**
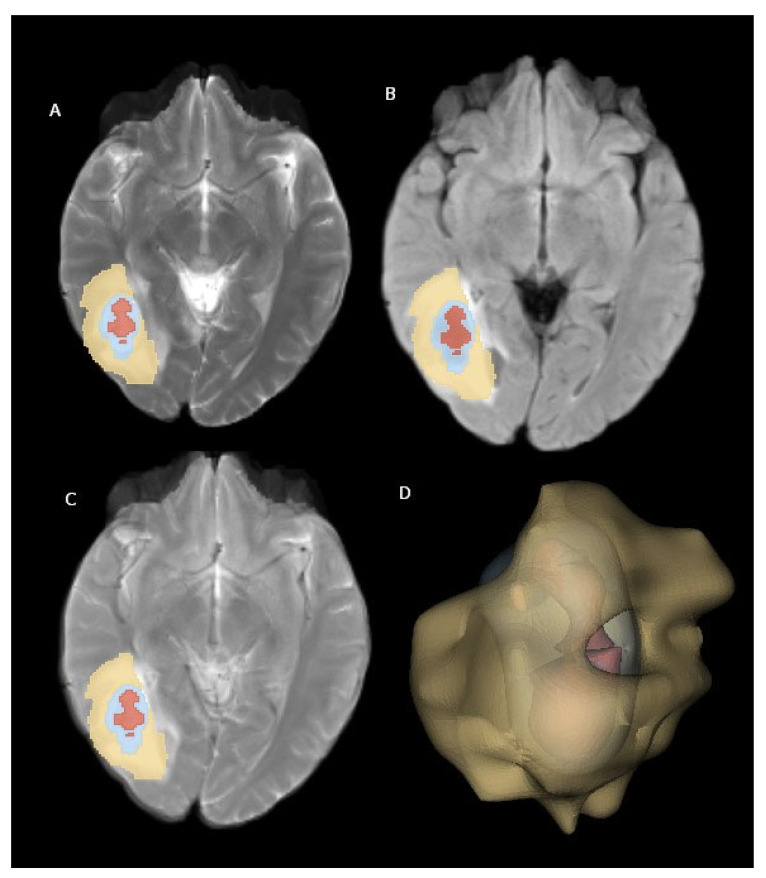
Brain metastasis from NSCLC located in right cerebral hemisphere. (**A**) T2-weighted images, (**B**) FLAIR-weighted images, (**C**) T2-FLAIR digital subtraction images, and (**D**) three-dimensional volume rendering of mass. Yellow: perilesional edema area; blue: contrast-enhanced area; red: necrosis area.

Using VTK, a three-dimensional model was constructed for each tumor region. These individual tumor models were then merged to create a composite representation. Finally, the generated 3D models were superimposed onto the corresponding MR images and visualized using Matplotlib (https://matplotlib.org/) and Seaborn (https://seaborn.pydata.org/). Figure 2 illustrates examples of brain metastasis in a patient diagnosed with non-small-cell lung cancer, rendered using the abovementioned 3D visualization technique.

### 2.3. Radiomic Feature Extraction

Radiomic features were extracted from the peritumoral edema area on T2-FLAIR digital subtraction images using the PyRadiomics 3.1.0 library (https://radiomics.github.io/pyradiomics.html (1 August 2024)) [19]. This process aims to characterize the edema based on quantitative image features.

The feature extraction process encompassed five filter types (original, Laplacian of Gaussian, logarithm, exponential, and wavelet) and three feature classes (first-order, texture, and shape) applied to the perilesional edema area (Figure 2). The filters and feature classes selected to characterize the peritumoral edema region enable a detailed and accurate analysis of the area. The original filter serves as a reference by reflecting the basic features of the data. The Laplacian of Gaussian (LoG) filter enhances the edges and details in the image, making the boundaries of the perilesional edema more distinct. The logarithmic filter improves low-contrast regions and highlights small but important features, making it useful for capturing subtle changes in peritumoral edema. The exponential filter emphasizes rapidly changing features and brings out the irregular structures in the perilesional edema more clearly. The wavelet filter allows for an analysis of the image at different resolution levels, making it possible to examine multi-scale structures. As for feature classes, first-order features include basic statistical properties (e.g., intensity and contrast), texture features analyze the textural patterns and structure in the image, and shape features describe the geometric structures within the region. These filters and feature classes allow for the accurate and effective characterization of the peritumoral edema region, aiding the model in producing more precise and reliable results. This resulted in a total of 1130 features being extracted for each patient.

### 2.4. Feature Selection and Machine Learning

All processes were implemented using the open-access Python 3.8 programming language and its associated libraries [20]. Feature selection and model development were conducted exclusively using the training cohort. Prior to model development, a min–max normalization scaler (range = 0–1) was applied to the radiomics data. Collinearity analysis was performed using the Pearson correlation coefficient (r) test, with a threshold of |r| ≥ 0.8. In cases of high collinearity, only one of the correlated features was retained for subsequent analysis. The training cohort was then split into training and testing sets using an 8:2 ratio.

To address the class imbalance between small-cell lung cancer (SCLC) and non-small-cell lung cancer (NSCLC) groups in the binary classification models, the Synthetic Minority Oversampling Technique (SMOTE) algorithm was employed to balance the number of samples in each group.

The performance of the classifiers was evaluated and compared using the area under the receiver operating characteristic curve (AUC). Several other performance metrics, including the F1 score, MCC (Matthews Correlation Coefficient), sensitivity, specificity, and accuracy, were also calculated. The formulas for the metrics are provided below (TP, true positive; TN, true negative; FP, false positive; FN, false negative):

- Accuracy = (TP + TN)/(TP + TN + FP + FN);
- Specificity = TN/(TN + FP);
- Recall (Sensitivity) = TP/(TP + FN);
- F1-score = 2 × (Precision × Recall)/(Precision + Recall);
- MCC = (TP × TN − FP × FN)/√((TP + FP) × (TP + FN) × (TN + FP) × (TN + FN)).

The maximum relevance–minimum redundancy (mRMR) method was employed for dimensionality reduction. mRMR is a widely used feature selection technique, allowing for the identification of the most informative features while minimizing redundancy [21]. This method is particularly valuable in high-dimensional datasets, such as those encountered in radiomics, where the goal is to improve the performance of classification algorithms by selecting a relevant subset of features. mRMR balances two crucial criteria: relevance (which is measured by the mutual information between a feature and the target variable) and redundancy (which is quantified by the mutual information between pairs of features). This approach has demonstrated considerable success in the context of radiomics data analysis, especially in binary classification tasks such as differentiating between malignant and benign tumors. Through selecting the most relevant and non-redundant features, mRMR helps to enhance the performance of classifiers, leading to more accurate and robust predictions. A total of four features selected by mRMR were used in the subsequent machine learning phase. The reason for selecting four features in this study is that this number optimized the model’s accuracy while minimizing the risk of overfitting, that is, when compared to other numbers, selecting four features yielded the best overall performance in both the training and validation sets. This choice allowed us to achieve statistically significant results; however, adding more features increased the complexity of the model, negatively affecting its accuracy.

In our study, there are various reasons for choosing the Naive Bayes method as a classification algorithm. First of all, the Naive Bayes algorithm is a simple and efficient algorithm that can produce rapid and effective results, especially in large-sized datasets [22]. Radiomics data are usually large in size as they contain a large number of properties. In this context, Naive Bayes’ low calculation costs and fast learning ability can accelerate the model development process, providing a practical advantage.

The Naive Bayes algorithm can show surprisingly good classification performance on large-sized datasets, although the features are based on the assumption of independence [23]. Although there is some correlation between the properties in radiomics data, it has been shown that Naive Bayes is resistant against this phenomenon, to a certain extent, and can produce acceptable results even in complex datasets [24]. Therefore, it is considered as an appropriate approach to cope with the high number of features encountered in radiomic analyses, as well as possible dependence problems between these features.

The interpretability of the Naive Bayes algorithm was another reason why it was chosen. It is relatively easier to understand the decision-making mechanisms of the Naive Bayes model through the use of methods such as SHAP (Shapley Additive Explanations) and to determine the relative importance of features [25]. This may contribute to the model’s clinically significant insights and the biological interpretation of radiomic properties.

For classification tasks (e.g., tumor grading and lesion detection), the Naive Bayes (NB) algorithm, as a probabilistic approach, has been widely used. NB is based on Bayes’ Theorem and calculates the probability of a sample belonging to a specific class based on the probabilities of the features observed in that class. The core (‘naive’) assumption of the algorithm is that all features are conditionally independent of each other under the given class label. Although this assumption may not always hold true in the real world, it significantly simplifies the associated computations. Thus, the primary reasons for choosing Naive Bayes in this study are its computational efficiency, its generally good performance in high-dimensional feature spaces (i.e., when there are many features), its ability to produce reasonable results with few samples, and its frequent use in the literature as a successful baseline model for similar problems. Despite the potential dependencies among the features, NB was determined to be an effective classifier for this task.

### 2.5. Interpreting the Machine Learning Model with SHAP

The Shapley Additive Explanations (SHAP) approach was employed to investigate the contributions of individual radiomic features and enhance the explainability of the machine learning model. SHAP values provide insights into the model’s decision-making process and help to identify influential features.

In particular, SHAP values quantify the marginal contribution of each feature to the model’s output across various feature combinations, ensuring a fair and unbiased assessment of feature importance. Through calculating the average marginal contribution of each feature across all possible combinations, the SHAP method provides a consistent and interpretable framework for evaluating feature contributions. This approach facilitates a deeper understanding of the model’s decision mechanism, enables a more comprehensive evaluation of its performance, and highlights key features that could be targeted for potential model improvements.

### 2.6. Statistical Analysis

Statistical analyses were performed using SPSS Statistics version 25.0 (IBM Inc., Armonk, NY, USA). Descriptive statistics for continuous variables are expressed as the mean ± standard deviation if the data followed a normal distribution. For non-normally distributed continuous variables, the median is reported. The Mann–Whitney U-test was employed to compare continuous variables that did not conform to a normal distribution with two-level categorical variables. Relationships between categorical variables were assessed using either Chi-square analysis or Fisher’s exact test depending on the expected cell frequencies. The discriminative ability of the machine learning models was evaluated using the area under the receiver operating characteristic curve (AUC). A *p*-value less than 0.05 was considered statistically significant for all analyses.

## 3. Results

The study included 109 NSCLC patients and 27 SCLC patients. The mean age of SCLC patients was 63.4 ± 11.7 years, and the mean age of NSCLC patients was 64.6 ± 10.3 years, with no significant difference between the two groups. Similarly, there were no significant differences in gender or history of extranodal metastasis at the time of diagnosis. Further details of the demographic data are presented in Table 1.

**Figure 3:**
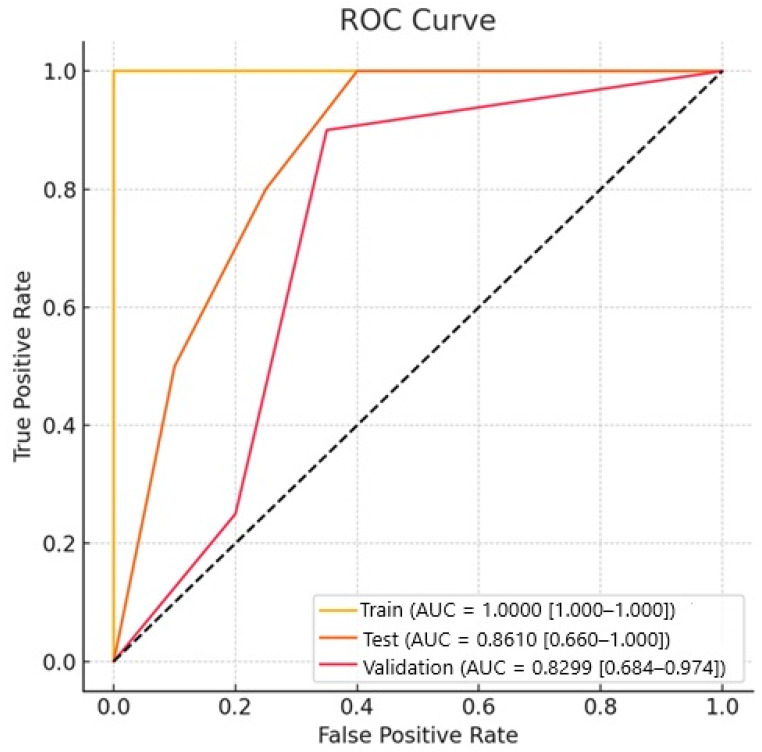
The receiver operating characteristic (ROC) curves for the model.

**Figure 4:**
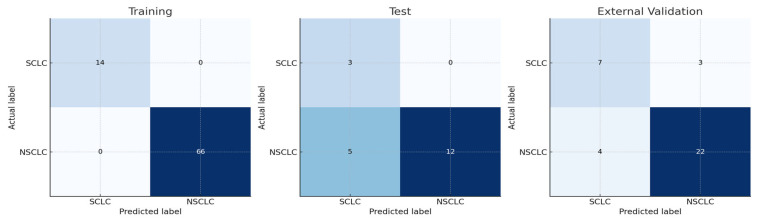
Confusion matrices for SCLC vs. NSCLC in the training, test, and external validation cohorts.

In our study, for the Naive Bayes model, the AUC value for NSCLC–SCLC discrimination in the test group was 0.861, and the AUC value for external validation was 0.829. The success of other models is shown in Table 2. The ROC curves for the groups are presented in Figure 3, while confusion matrices are shown in Figure 4.

**Figure 5:**
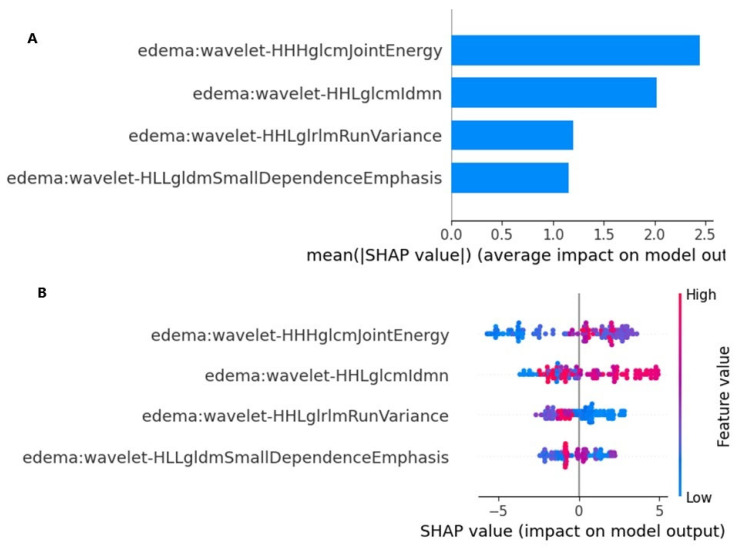
SHAP analysis: (**A**) SHAP Bar Plot. This graph shows the most important features in terms of the predictions of our model. The feature with the greatest effect is wavelet-HHHglcmJointEnergy, with a SHAP value of approximately 2.5. These features explain most of the variation in the model’s forecasts. (**B**) SHAP summary plot. This summary graph shows the effect of each feature on the model outputs and the distribution of the values of these features.

The SHAP values provided a quantitative explanation for the Naive Bayes method. SHAP bar plot graphs and summary graphs visually and succinctly represent the importance of the range and distribution of radiomics features on the model’s output, relating the feature’s value to its impact. From Figure 5A,B, we observed that the wavelet-HHHglcmJointEnergy feature was the radiomics feature contributing the most to the model overall. The force plot (Figure 5) allows for the interpretation of the evaluation of a single patient. It visualizes each feature’s SHAP value as a force that either increases or decreases the evaluation, with each prediction starting from the base value (–0.12), namely, the average SHAP value of all predictions. The length of the arrow indicates how much (in percentage) a particular feature’s SHAP value contributes to the force.

**Figure 6:**
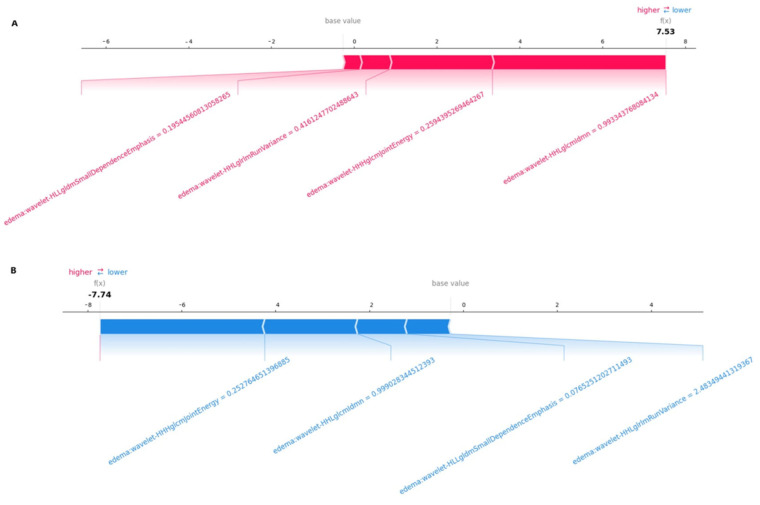
A SHAP force plot, which interprets the evaluation of individual patients by visualizing each feature’s SHAP value as a force that either increases or decreases the evaluation. Each prediction starts from the base value (−0.12), which is the average SHAP value of all predictions. The length of the arrow indicates the percentage contribution of a feature’s SHAP value, while the color represents whether its contribution is positive (red) or negative (blue). (**A**) This patient’s SHAP value is 7.53, which is higher than the base value (−0.12), indicating that we could evaluate this patient as having SCLC brain metastasis. Among the features, the positive (red) wavelet-HHLglcmgcmldmn arrow (with a value of 0.99) and the positive (red) wavelet-HLLgldmSmallDependenceEmphasis arrow (with a value of 0.19) indicate the significant contributions of these features to the evaluation of SCLC brain metastasis. (**B**) For another patient, the SHAP value is −7.74, which is lower than the base value (−0.12). Therefore, we could evaluate this patient as belonging to the brain metastasis group. In this patient, the wavelet-HHHglcmJointEnergy arrow, with a value of −0.25, made a negative (blue) contribution, facilitating the evaluation of NSCLC brain metastasis.

The color of the arrow represents whether the contributions were positive (red) or negative (blue). As shown in Figure 6A, this patient’s SHAP value was 7.53, which was higher than the base value (−0.12), indicating that we could evaluate this patient as having SCLC brain metastasis. Among these features, the positive (red) wavelet-HHLglcmgcmldmn arrow (with a value of 0.99) and the positive (red) wavelet-HLLgldmSmallDependenceEmphasis arrow (with a value of 0.19) significantly contributed to the evaluation of SCLC brain metastasis. As shown in Figure 6B, for another patient, the SHAP value was −7.74, which was lower than the base value (−0.12). Therefore, we could evaluate this patient as belonging to the NSCLC brain metastasis group. In this patient, the wavelet-HHHglcmJointEnergy arrow (with a value of −0.25) made a negative (blue) contribution, thus aiding in the evaluation of NSCLC brain metastasis.

## 4. Discussion

Knowing the histologic type of patients with brain metastases is crucial for determining an appropriate treatment strategy. From this perspective, our study aimed to investigate whether NSCLC and SCLC can be differentiated using an artificial intelligence-based approach in patients with lung cancer metastases. We created digital subtraction images to quantify the mismatch between T2-FLAIR images of the peritumoral edema area. The findings revealed that NSCLC–SCLC discrimination can be performed with high accuracy, as evidenced by AUC values of 0.861 in the test group and 0.829 in the validation group. Studies have reported high rates for the detection of the histological type of brain metastasis with artificial intelligence using different imaging findings relating to the primary tumor [26,27]. Considering that radiomic data of the primary tumor are useful in determining the histological subtype, we considered that the peritumoral edema area may be useful in differentiating histological types.

Peritumoral edema in patients with lung cancer brain metastasis can aid in predicting the histologic subtype. Studies have shown that the peritumoral edema area in patients with lung cancer and brain metastasis is smaller in SCLC patients than in NSCLC patients, and it can be used in predicting survival [28]. It has also been demonstrated that a larger peritumoral edema area in brain metastasis case can serve as a negative predictive parameter for both the response to radiotherapy and survival. The mechanisms of peritumoral edema formation in primary brain tumors and brain metastases are different. Basically, differences in the perilesional edema between high-grade glial tumors and metastases may result from differences in vascular permeability and angiogenesis processes in the tumor microenvironment. In glial tumors, VEGF and other angiogenic factors produced by tumor cells disrupt the blood–brain barrier in normal brain tissue and lead to the formation of the edema [29]. Meanwhile, in metastases, a vasogenic edema occurs as a result of the disruption of the blood–brain barrier [30]. In a study on the structure of peritumoral edema caused by primary brain tumors and brain metastases, it was reported that multiple factors are involved in the associated etiopathogenesis [31]. Although Toch et al. [32] suggested that the structure of peritumoral edema may be the result of the dysfunction of the glymphatic system, the exact mechanism remains unclear.

Peritumoral edema is hyperintense on T2 and FLAIR images in both primary and metastatic tumors. In both cases, there is a specific marker that can distinguish between the two on conventional brain MR imaging. Demirel et al. [11] demonstrated that artificial intelligence-based models using radiomics data obtained from the peritumoral edema area on T2-FLAIR digital subtracted images can differentiate high-grade glial tumors from brain metastases. As is well known, the T2-FLAIR mismatch finding is considered to be a highly specific indicator, especially in primary glial tumors, and especially for detecting IDH mutant-1p19q non-codeleted patients [33,34]. The mechanisms underlying the T2-FLAIR imaging phenomenon are complex and involve several factors. 

The molecular etiopathogenesis of the T2-FLAIR mismatch sign is the result of a complex situation of large-scale metabolic, epigenetic, and microenvironmental changes caused by the IDH mutation [33,34]. However, in cases of peritumoral edema, the accepted mechanism of edema development in metastases is insufficient to explain the success of our model, and there are several hypotheses that may explain this issue. As is well known, the primary biological behaviors of NSCLC and SCLC tumors differ. Rapid growth and necrosis in SCLC may lead to an altered water content and more pronounced peritumoral edema, which may result in a different appearance on T2-FLAIR imaging. Meanwhile, NSCLC metastases may present a more organized growth pattern, resulting in less peritumoral edema [35]. The vascularity of tumors may also influence the amount and type of peritumoral edema seen in brain metastases.

SCLC is generally associated with a higher degree of vascularity and greater disruption of the blood–brain barrier (BBB) than NSCLC, leading to more significant vasogenic edema around the tumor. Therefore, T2-FLAIR mismatch may be a result of these different edema patterns [36]. The degree of necrosis in metastases may also present differences. SCLC is more likely to show central necrosis due to rapid tumor growth and insufficient blood supply. These histopathologic differences may contribute to the differences in the T2-FLAIR mismatch sign between these two types of brain metastases [37]. The tumor microenvironment may be another factor that can explain this phenomenon. SCLC tumors may have a hypoxic microenvironment and more necrosis due to their rapid growth, leading to changes in water content and edema patterns which are detectable in T2 and FLAIR images [35]. As another hypothesis, the molecular composition of the tumor and its microenvironment may also influence the imaging characteristics observed in T2-FLAIR sequences. SCLC and NSCLC differ in terms of the expression of various molecules related to angiogenesis, tumor progression, and treatment response. In lung cancer patients, targeted therapies directed at molecular mutations identified in both SCLC and NSCLC have been shown to contribute to improved survival outcomes [38,39,40]. These mutations exhibit similar behavioral patterns not only in the primary tumor, but also in metastatic lesions. Accordingly, these molecular alterations may drive uncontrolled cell proliferation and accelerate metastatic spread. Such mutations could lead to more rapid development of peritumoral edema and induce structural changes in the surrounding tissue. In particular, the molecular environment in SCLC promotes more aggressive progression, characterized by increased production of inflammatory cytokines and alterations in the extracellular matrix. These changes may underlie the differences in the structure of peritumoral edema detectable by T2-FLAIR imaging. Digital subtraction can help to differentiate tumor types by making these differences more prominent.

As our study was performed with an open dataset and data obtained from our own institution, a detailed analysis of the subtypes of non-small-cell lung cancers could not be performed. Peritumoral edema is known to be more aggressive in SCLC patients than in NSCLC patients. However, it is also known that brain metastasis-associated edema is more aggressive in adenocarcinoma patients with mutations such as EGFR or ALK when compared to other NSCLC types [41]. In this regard, our study may inspire future studies on whether subtyping can be performed from peritumoral edema, especially in patients with EGFR or ALK mutations.

This preliminary study provides evidence that T2-FLAIR digital subtraction images can be used for histologic subtyping in lung cancer patients with brain metastases through an artificial intelligence-based approach. We believe that radiologic images will be useful in illuminating certain situations using radiomics data at the micro level, along with their gross guiding properties. In particular, future cellular-based investigations are needed to uncover the reasons for the observed structural differences. Furthermore, the different peritumoral edema structures associated with histologic subtypes of lung cancer, as observed in current radiologic images, may contribute to the re-evaluation of treatment options, prognosis, and survival prediction. We hope that this study will inspire multicenter studies with large samples, or even post-mortem investigations.

Our study has some limitations. First, it was retrospective, and the number of patients was relatively small. The patients included in the study were only divided into two groups (small-cell and non-small-cell lung cancer), as the non-small-cell subtypes were not homogeneously distributed. Another limitation of our study was that there was insufficient information about the demographic data of the patients in the data obtained from open sources, and no additional gene expression or molecular information was known regarding the histopathologic subtypes. As the treatment strategy for lung cancer is typically determined according to the small-cell vs. non-small-cell characterization, these limitations were ignored.

## 5. Conclusions

T2-FLAIR digital subtraction images of the peritumoral edema area can be used to differentiate NSCLC and SCLC in patients with lung cancer brain metastases. Although brain metastasis-associated edema appears similar in terms of gross appearance on MR imaging, it contains more information than what is immediately visible. This study has the potential to inspire future studies that not only focus on determining the subtypes of lung cancer metastases but also explore whether histological typing can be performed in brain metastases of other cancer types. Postmortem studies will provide more detailed data on the microstructural characteristics of the peritumoral edema area.

## Figures and Tables

**Table 1:** Demographic data (SCLC: small-cell lung carcinoma; NSCLC: non-small-cell lung carcinoma).

	SCLC (*n* = 27)	NSCLC (*n* = 109)	*p*
Age (years)	63.4 ± 11.7	64.6 ± 10.3	0.665
Gender			0.730
Male	12 (70%)	55 (66.3%)	
Female	5 (30%)	28 (33.7%)	
Extranodal Metastasis	7 (41.2%)	28 (34.6%)	0.600

**Table 2:** Details of model performance. NB: Naive Bayes; AUC: area under the curve; ACC: accuracy; SENS: sensitivity; SPEC: specificity; MCC: Matthews Correlation Coefficient.

NB Model	AUC	ACC	F1	SENS	SPEC	MCC
Train *n* = 80	1	1	1	1	1	1
	(1.000–1.000)	(1.000–1.000)	(1.000–1.000)	(1.000–1.000)	(1.000–1.000)	(1.000–1.000)
Test (*n* = 20)	0.861	0.694	0.62	0.9	0.615	0.524
	(0.660–1.000)	(0.463–0.925)	(0.436–0.825)	(0.789–1.000)	(0.347–0.883)	(0.480–0.563)
External Validation (*n* = 36)	0.829	0.794	0.8	0.875	0.722	0.569
	(0.684–0.974)	(0.630–0.958)	(0.634–0.913)	(0.742–1.000)	(0.545–0.899)	(0.485–0.712)

## Data Availability

The anonymized data collected in this study are available as open data via the TCIA online data repository: https://doi.org/10.1038/s41597-024-03021-9 (https://www.nature.com/articles/s41597-024-03021-9), accessed on 1 April 2020, and https://doi.org/10.1007/s10278-013-9622-7 (https://link.springer.com/article/10.1007/s10278-013-9622-7), accessed on 1 April 2020.

## References

[1] Thai A., Solomon B., Sequist L.V., Ganior J.F., Heist R.S. (2021). Lung cancer. Lancet.

[2] Karachaliou N., Pilotto S., Lazzari C., Bria E., de Marinis F., Rosell R. (2016). Cellular and Molecular Biology of Small Cell Lung Cancer: An Overview. Transl. Lung Cancer Res..

[3] Kalemkerian G.P., Loo B.W., Akerley W., Attia A., Boumber Y., Decker R., Dobelbower M., Dowlati A., Downey R.J., Ettinger D.S. (2018). NCCN Guidelines Insights: Small Cell Lung Cancer, Version 2.2018. J. Natl. Compr. Canc. Netw..

[4] Dela Cruz C.S., Tanoue L.T., Matthay R.A. (2011). Lung Cancer: Epidemiology, Etiology, and Prevention. Clin. Chest Med..

[5] Mithoowani H., Febbraro M. (2022). Non-Small-Cell Lung Cancer in 2022: A Review for General Practitioners in Oncology. Curr. Oncol..

[6] Manapov F., Käsmann L., Roengvoraphoj O., Dantes M., Schmidt-Hegemann N.S., Belka C., Eze C. (2018). Prophylactic cranial irradiation in small-cell lung cancer: Update on patient selection, efficacy and outcomes. Lung Cancer.

[7] Zeng H., Zheng D., Witlox W.J.A., Levy A., Traverso A., Kong F.S., Houben R., De Ruysscher D.K.M., Hendriks L.E.L. (2022). Risk factors for brain metastases in patients with small cell lung cancer: A systematic review and meta-analysis. Front. Oncol..

[8] Zheng M. (2016). Classification and pathology of lung cancer. Surg. Oncol. Clin. N. Am..

[9] Li Y., Yu R., Chang H., Yan W., Wang D., Li F., Cui Y., Wang Y., Wang X., Yan Q. (2024). Identifying Pathological Subtypes of Brain Metastasis from Lung Cancer Using MRI-Based Deep Learning Approach: A Multicenter Study. J. Imaging Inform. Med..

[10] Bozdağ M., Er A., Çinkooğlu A. (2021). Histogram analysis of ADC maps for differentiating brain metastases from different histological types of lung cancers. Can. Assoc. Radiol. J..

[11] Nardone V., Nanni S., Pastina P., Vinciguerra C., Cerase A., Correale P., Guida C., Giordano A., Tini P., Reginelli A. (2019). Role of perilesional edema and tumor volume in the prognosis of non-small cell lung cancer (NSCLC) undergoing radiosurgery (SRS) for brain metastases. Strahlenther. Onkol..

[12] Arrieta O., Bolaño-Guerra L.M., Caballé-Pérez E., Lara-Mejía L., Turcott J.G., Gutiérrez S., Lozano-Ruiz F., Cabrera-Miranda L., Arroyave-Ramírez A.M., Maldonado-Magos F. (2023). Perilesional edema diameter associated with brain metastases as a predictive factor of response to radiotherapy in non-small cell lung cancer. Front. Oncol..

[13] Kniep H.C., Madesta F., Schneider T., Hanning U., Schönfeld M.H., Schön G., Stark A.M., Broocks G., Fiehler J., Gellissen S. (2019). Radiomics of Brain MRI: Utility in Prediction of Metastatic Tumor Type. Radiology.

[14] Zhang J., Jin J., Ai Y., Zhang F., Liu B. (2021). Differentiating the Pathological Subtypes of Primary Lung Cancer for Patients with Brain Metastases Based on Radiomics Features from Brain CT Images. Eur. Radiol..

[15] Demirel E., Dilek O. (2025). Utilizing Radiomics of Peri-Lesional Edema in T2-FLAIR Subtraction Digital Images to Distinguish High-Grade Glial Tumors From Brain Metastasis. J. Magn. Reason. Imaging.

[16] Clark K., Vendt B., Smith K., Freymann J., Kirby J., Koppel P., Moore S., Phillips S., Maffitt D., Pringle M. (2013). The Cancer Imaging Archive (TCIA): Maintaining and operating a public information repository. J. Digit. Imaging.

[17] Sarthak P., Ashish S., Saima R., Aimilia G., Mark B., Phuc N., Sung M.H., Dimitrios B., James M., Grayson M. (2020). The Cancer Imaging Phenomics Toolkit (CaPTk): Technical Overview. Brainlesion.

[18] Yaniv Z., Lowekamp B.C., Johnson H.J., Beare R. (2018). SimpleITK image-analysis notebooks: A collaborative environment for education and reproducible research. J. Digit. Imaging.

[19] van Griethuysen J.J.M., Fedorov A., Parmar C., Hosny A., Aucoin N., Narayan V., Beets-Tan R.G.H., Fillion-Robin J.-C., Pieper S., Aerts H.J.W.L. (2017). Computational radiomics system to decode the radiographic phenotype. Cancer Res..

[20] Abraham A., Pedregosa F., Eickenberg M., Gervais P., Mueller A., Kossaifi J., Gramfort A., Thirion B., Varoquaux G. (2014). Machine learning for neuroimaging with scikit-learn. Front. Neuroinform..

[21] Gu X., Guo J., Xiao L., Li C. (2022). Conditional mutual information-based feature selection algorithm for maximal relevance minimal redundancy. Appl. Intell..

[22] Peretz O., Koren M., Koren O. (2024). Naive Bayes classifier–An ensemble procedure for recall and precision enrichment. Eng. Appl. Artif. Intell..

[23] Yang F.J. (2018). An implementation of naive bayes classifier. Proceedings of the 2018 International Conference on Computational Science and Computational Intelligence (CSCI).

[24] Chen S., Webb G.I., Liu L., Ma X. (2020). A novel selective naïve Bayes algorithm. Knowl.-Based Syst..

[25] Molnar C. (2020). Interpretable Machine Learning.

[26] Demirel E., Özer G.Ç., Dilek O., Özdemir Ç., Boyacı M.G., Korkmaz S. (2021). Differential diagnosis of glioblastoma and solitary brain metastasis–the success of artificial intelligence models created with radiomics data obtained by automatic segmentation from conventional MRI sequences. Ceska Slov. Neurol. Neurochir..

[27] Cho S.J., Sunwoo L., Baik S.H., Bae Y.J., Choi B.S., Kim J.H. (2021). Brain metastasis detection using machine learning: A systematic review and meta-analysis. Neuro-Oncology.

[28] Komatsu T., Kunieda E., Oizumi Y., Tamai Y., Akiba T. (2013). Clinical characteristics of brain metastases from lung cancer according to histological type: Pretreatment evaluation and survival following whole-brain radiotherapy. Mol. Clin. Oncol..

[29] Arvanitis C.D., Ferraro G.B., Jain R.K. (2020). The blood–brain barrier and blood–tumour barrier in brain tumours and metastases. Nat. Rev. Cancer.

[30] Mo F., Pellerino A., Soffietti R., Rudà R. (2021). Blood–Brain Barrier in Brain Tumors: Biology and Clinical Relevance. Int. J. Mol. Sci..

[31] Solar P., Hendrych M., Barak M., Valekova H., Hermanova M., Jancalek R. (2022). Blood-Brain Barrier Alterations and Edema Formation in Different Brain Mass Lesions. Front. Cell Neurosci..

[32] Toh C.H., Siow T.Y., Castillo M. (2021). Peritumoral brain edema in metastases may be related to glymphatic dysfunction. Front. Oncol..

[33] Jain R., Johnson D.R., Patel S.H., Castillo M., Smits M., van den Bent M.J., Chi A.S., Cahill D.P. (2020). “Real world” use of a highly reliable imaging sign:“T2-FLAIR mismatch” for identification of IDH mutant astrocytomas. Neuro-Oncology.

[34] Pinto C., Noronha C., Taipa R., Ramos C. (2022). T2-FLAIR mismatch sign: A roadmap of pearls and pitfalls. Br. J. Radiol..

[35] Waqar S.N., Samson P.P., Robinson C.G., Bradley J., Devarakonda S., Du L., Govindan R., Gao F., Puri V., Morgensztern D. (2018). Non-small-cell Lung Cancer With Brain Metastasis at Presentation. Clin. Lung Cancer.

[36] Kiyose M., Herrmann E., Roesler J., Zeiner P.S., Steinbach J.P., Forster M.T., Plate K.H., Czabanka M., Vogl T.J., Hattingen E. (2023). MR imaging profile and histopathological characteristics of tumour vasculature, cell density and proliferation rate define two distinct growth patterns of human brain metastases from lung cancer. Neuroradiology.

[37] Yoo J., Cha Y.J., Park H.H., Park M., Joo B., Suh S.H., Ahn S.J. (2022). The Extent of Necrosis in Brain Metastases May Predict Subtypes of Primary Cancer and Overall Survival in Patients Receiving Craniotomy. Cancers.

[38] Boldig C., Boldig K., Mokhtari S., Etame A.B. (2024). A Review of the Molecular Determinants of Therapeutic Response in Non-Small Cell Lung Cancer Brain Metastases. Int. J. Mol. Sci..

[39] Zhu Y., Cui Y., Zheng X., Zhao Y., Sun G. (2022). Small-Cell Lung Cancer Brain Metastasis: From Molecular Mechanisms to Diagnosis and Treatment. Biochim. Biophys. Acta Mol. Basis Dis..

[40] Yousefi M., Bahrami T., Salmaninejad A., Molaei F., Baradaran B. (2017). Lung Cancer-Associated Brain Metastasis: Molecular Mechanisms and Therapeutic Options. Cell Oncol..

[41] Wen J., Yu J.Z., Liu C., Ould Ismail A.A.O., Ma W. (2024). Exploring the Molecular Tumor Microenvironment and Translational Biomarkers in Brain Metastases of Non-Small-Cell Lung Cancer. Int. J. Mol. Sci..

